# Superior Mesenteric Artery Thrombosis in COVID-19-Positive Patients: A Rare Coincidence

**DOI:** 10.7759/cureus.62136

**Published:** 2024-06-11

**Authors:** Rushabh Parekh, Virendra Athavale, Saili Kelshikar

**Affiliations:** 1 General Surgery, Dr. D. Y. Patil Medical College, Hospital and Research Centre, Pune, IND

**Keywords:** surgical emergencies, acute abdomen in covid-19, bowel ischemia, covid-19, superior mesenteric artery thrombosis

## Abstract

Since the start of the SARS-CoV-2 pandemic, which is otherwise known as the worldwide coronavirus disease, 2019, has had a well-established pro-thrombotic character. Patients often first exhibit respiratory symptoms, and those whose severity increases eventually develop acute hypoxic respiratory failure. The systemic hypercoagulable condition and arterial/venous thrombosis related to COVID-19 have a poor prognosis. Even though superior mesenteric artery (SMA) thrombosis and acute mesenteric ischemia (AMI) are uncommon, they frequently coexist with fatal gastrointestinal (GI) pathologies that necessitate prompt diagnosis and treatment by the doctor. This calls for more research into the effects of anticoagulation therapy in COVID-19-positive patients. The main treatment aims for this condition are early detection, surgical or intravascular re-establishment of blood supply to the ischemic bowel, and surgical resection. The study aimed to see the outcome after surgical intervention in patients with SMA thrombosis post-COVID-19 infection. This study was from March 2021 to January 2022, with a sample size of 5 patients with SMA thrombosis, which was confirmed on contrast-enhanced computed tomography (CECT) abdomen and pelvis with angiography. The patients underwent exploratory laparotomy. Bowel resection and anastomosis were performed in three individuals; bowel resection and stoma placement were performed in two patients. Doctors have significant clinical challenges as a result of the thromboembolic manifestations of the unexpected and deadly nature of the virus, such as AMI. The high morbidity and mortality associated with AMI calls for further study on prophylactic anticoagulation therapy in COVID-19-positive individuals.

## Introduction

COVID-19 is a unique respiratory virus that mostly spreads through respiratory droplets, which led to a pandemic that affected the whole world. Primary occurrences took place in Wuhan, China, more than four years ago, when patients originally came with pneumonia of unclear origin and respiratory symptoms [1].

The abdominal organs are supplied by the celiac artery (CA), superior mesenteric artery (SMA), and inferior mesenteric artery (IMA). A defense against ischemia is the extensive collateralization between splanchnic arteries. The CA and SMA are anastomose with the inferior and superior pancreaticoduodenal arteries. Rarely is the arc of Buhler used to join these two primary vessels. There are three significant anastomoses between the SMA and IMA: the Riolan arc, the central artery, and the marginal artery of Drummond. The IMA forms anastomoses with the femoral, internal, and external iliac arteries, as well as the aorta. The collateral blood supply protects the bowel and provides defense against ischemia. Even when the extramural artery supply is severely compromised, a network of intramural submucosal arteries aids in the preservation of certain bowel segments. Additionally, ischemia causes a redistribution of intramural blood flow that provides mucosa preservation. It has been proven that the bowel can go up to 12 hours with a 75% reduction in blood flow without suffering any serious harm [2].

Acute mesenteric ischemia (AMI) is uncommon; however, the high fatality rate can be attributed to the AMI's nonspecific signs and symptoms. It is common to see *pain out of proportion to physical examination findings* during the preliminary stages of ischemia when the abdomen is soft yet tender. With rebound guarding, bowel infarction causes distention and excruciating agony. The main therapy foci for this condition are early detection and blood flow restoration to the ischemic bowel, surgical resection of the gangrenous bowel, and postoperative management [3]. COVID-19 is usually related to coagulopathy and thrombotic complications. Only a few case studies have discussed the uncommon thrombotic consequence known as AMI, which has a significant death and morbidity rate. Reduced blood supply to the bowel causes intestinal loop ischemia lesions and gangrene and can lead to death from peritonitis and septic shock [4].

## Case presentation

Case 1

**Figure 1:**
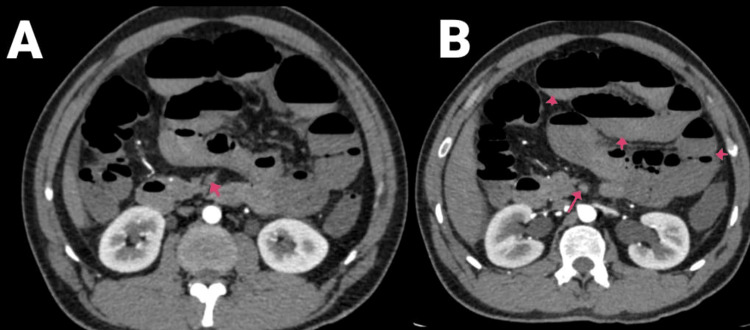
(A and B) CECT of the abdomen and pelvis with angiography of the patient (arrows showing SMA thrombosis and air-fluid levels). CECT, contrast-enhanced computed tomography; SMA, superior mesenteric artery

**Figure 2:**
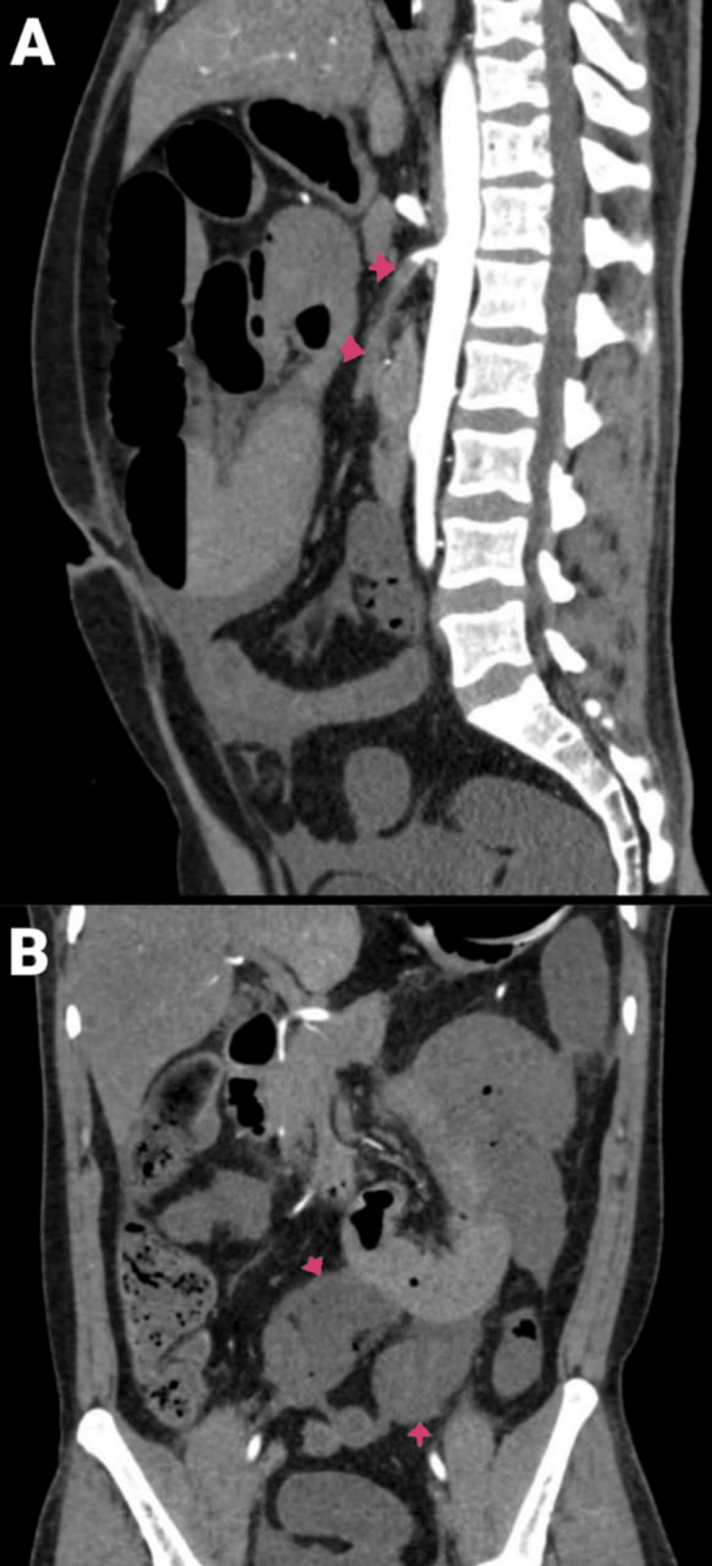
CECT of the abdomen and pelvis with angiography of the patient: (A) sagittal section and (B) coronal section. Arrows are showing SMA thrombosis with dilated jejunal and proximal ileal loops. CECT, contrast-enhanced computed tomography; SMA, superior mesenteric artery

A 27-year-old male had been complaining of pain in the abdomen for five days, associated with four episodes of vomiting and constipation. The patient was COVID-19-positive with no comorbidities, previous history of surgeries, or trauma. The CECT abdomen and pelvis report indicated thrombosis in the SMA and its jejunal and ileal branches, along with multiple dilated jejunal and proximal ileal loops due to bowel ischemia (Figures 1A-1B, 2A-2B).

Intraoperative Findings

**Figure 3:**
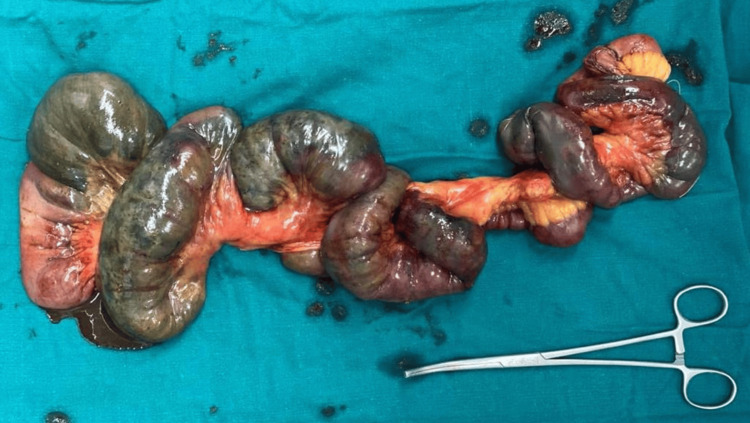
Resected specimen of the gangrenous bowel.

It was found that the small bowel was gangrenous for about 100 cm from the duodenojejunal flexure to 40 cm from the ileocecal junction, with the remaining gut and solid organs being healthy (Figure 3). Resection of the gangrenous bowel along with SMA thrombectomy was done with jejunoileal anastomosis.

Postoperative Management

The patient had no postoperative complications, was gradually started on a diet along with anticoagulation therapy, and was discharged. The patient had no complaints for one year.

Case 2

**Figure 4:**
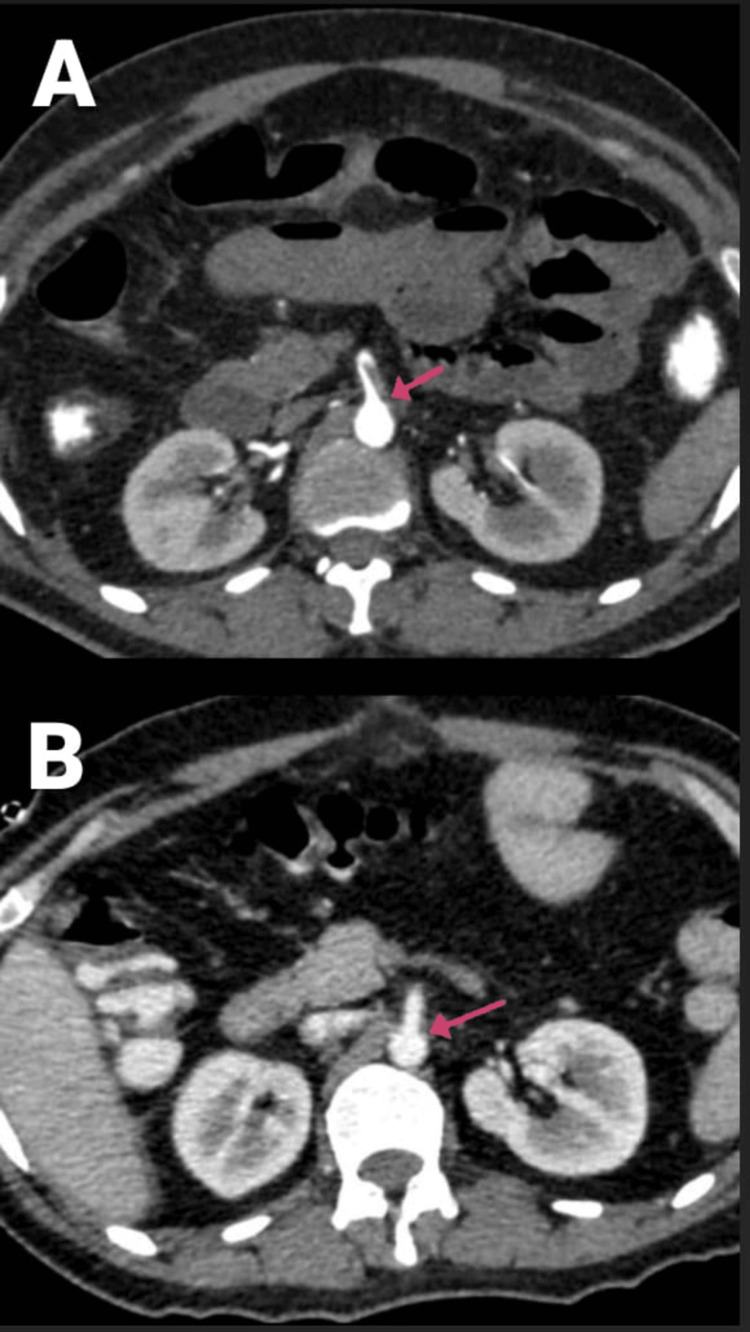
(A and B) CECT of the abdomen and pelvis with angiography of the patient (arrows showing a partial filling defect in the SMA along its posterior and left lateral wall). CECT, contrast-enhanced computed tomography; SMA, superior mesenteric artery

**Figure 5:**
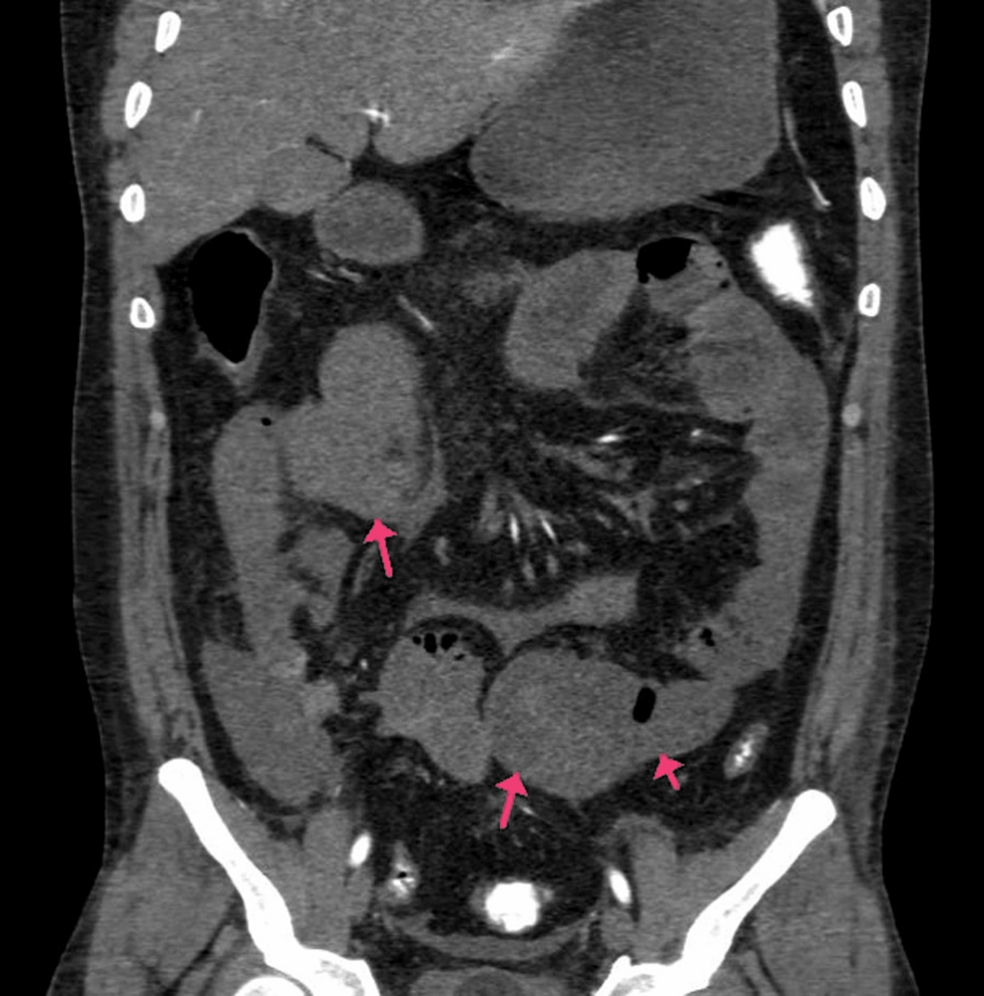
CECT of the abdomen and pelvis with angiography of the patient (arrows mild hypo-enhancement of the walls with a mildly dilated caliber of ileal loops and pneumatosis). CECT, contrast-enhanced computed tomography; SMA, superior mesenteric artery

A 29-year-old male had been complaining of abdominal pain for two days, associated with two to three episodes of vomiting and constipation. He is a known alcoholic. The patient was COVID-19-positive and had no other comorbidities or previous history of surgeries or trauma. On the CECT of the abdomen and pelvis, a partial filling defect is noted in the SMA along its posterior and left lateral wall for a length of about 4.5 cm from its origin, suggesting partial thrombosis (Figures 4A-4B, 5). The rest of the SMA and its branches showed normal contrast opacification. Mild hypo-enhancement of walls with mildly dilated caliber of ileal loops was seen with pneumatosis. No evidence of bowel perforation was present.

Intraoperative Findings

**Figure 6:**
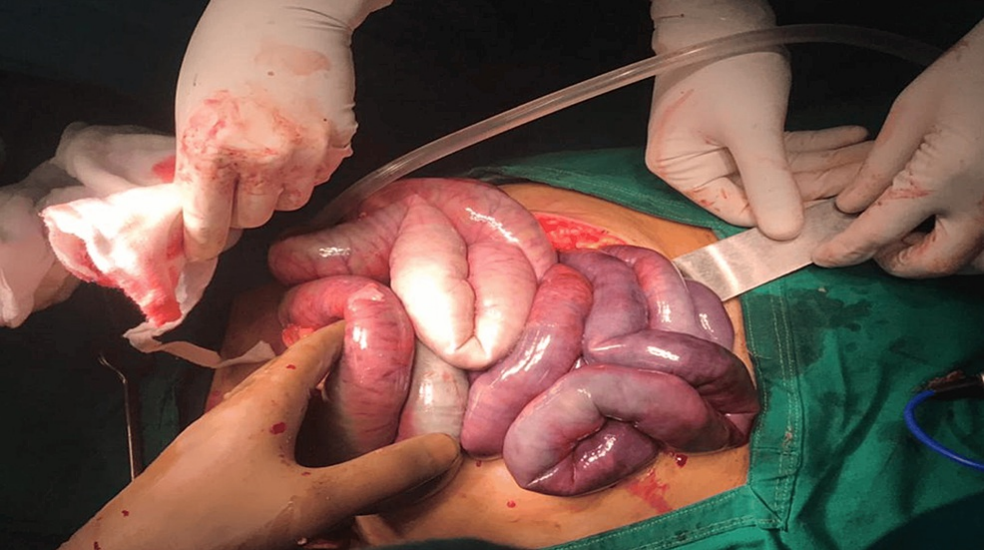
Intra-op photo of the gangrenous bowel.

The gangrenous small bowel was seen from about 150 cm from the duodenojejunal flexure to about 60 cm proximal to the ileocecal junction (Figure 6). Resection of the gangrenous small bowel without SMA thrombectomy and ileoileal anastomosis was done.

Postoperative Management

The patient suffered from hypovolemia and septic shock and was started on inotropic support and higher antibiotics. However, the patient succumbed two days post-op.

Case 3 

**Figure 7:**
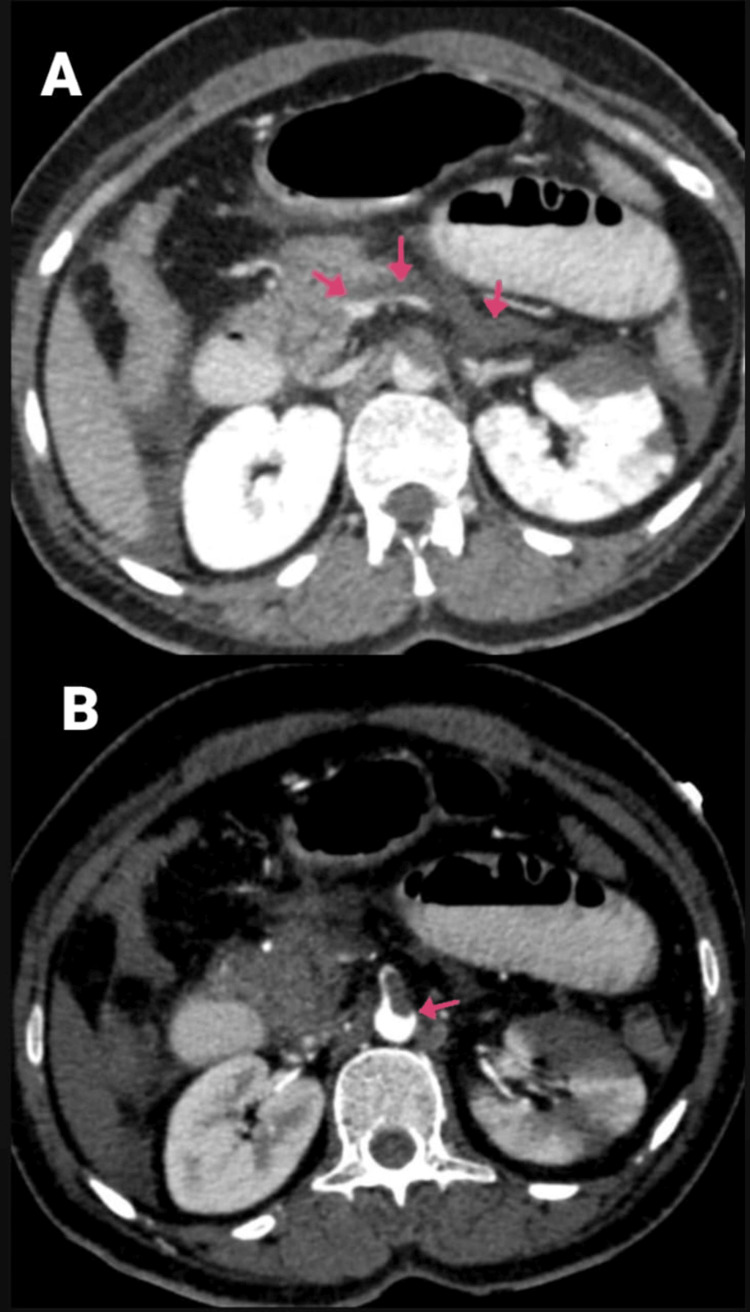
(A and B) CECT of the abdomen and pelvis with angiography of the patient (arrows showing the SMA thrombosis and thrombosed portal confluence). CECT, contrast-enhanced computed tomography; SMA, superior mesenteric artery

**Figure 8:**
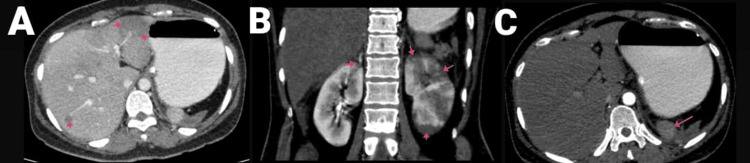
CECT of the abdomen and pelvis with angiography of the patient, with arrows showing (A) hepatic infarcts, (B) renal infarcts, and (C) shrunken spleen with splenic infarct. CECT, contrast-enhanced computed tomography

A 40-year-old female had been complaining of pain in the abdomen in the epigastric region and giddiness for one day. There were no complaints of vomiting or nausea, and she had a recently diagnosed case of diabetes mellitus. The patient was COVID-19-positive. CECT of the abdomen and pelvis showed the abdominal aorta at the level of the lower margin of the D12 vertebra to the L1 vertebra with a hypodense, non-enhancing filling defect measuring 2.5 cm in length, causing minimal narrowing of the lumen and involving the origin of the celiac trunk and SMA (Figures 7A-7B). The coeliac trunk and its branches, such as the left gastric, common hepatic, and splenic arteries, were not visualized and did not show any opacification with contrast agent likely thrombosis. A hypodense filling defect was noted at the origin of the SMA anterior to its origin and proximal portion and in its ileal branches, suggesting a thrombus. There was also evidence of infarcts in the kidney and liver. The spleen was shrunken along with a splenic infarct (Figures 8A-8C).

Intraoperative Findings

A gangrenous small bowel was seen from about 170 cm from the duodenojejunal flexure to about 60 cm distal to the ileocecal junction. Resection of the gangrenous bowel along with SMA thrombectomy and ileostomy was done.

Postoperative Management

The patient had no postoperative complications and was started on anticoagulation therapy, diet, and discharge. The patient complained of pain in the abdomen three months postoperatively and was managed conservatively.

Case 4

**Figure 9:**
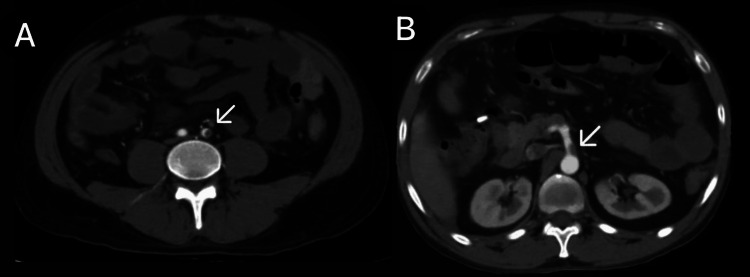
(A and B) CECT of the abdomen and pelvis with angiography of the patient (arrows showing the SMA thrombosis). CECT, contrast-enhanced computed tomography; SMA, superior mesenteric artery

**Figure 10:**
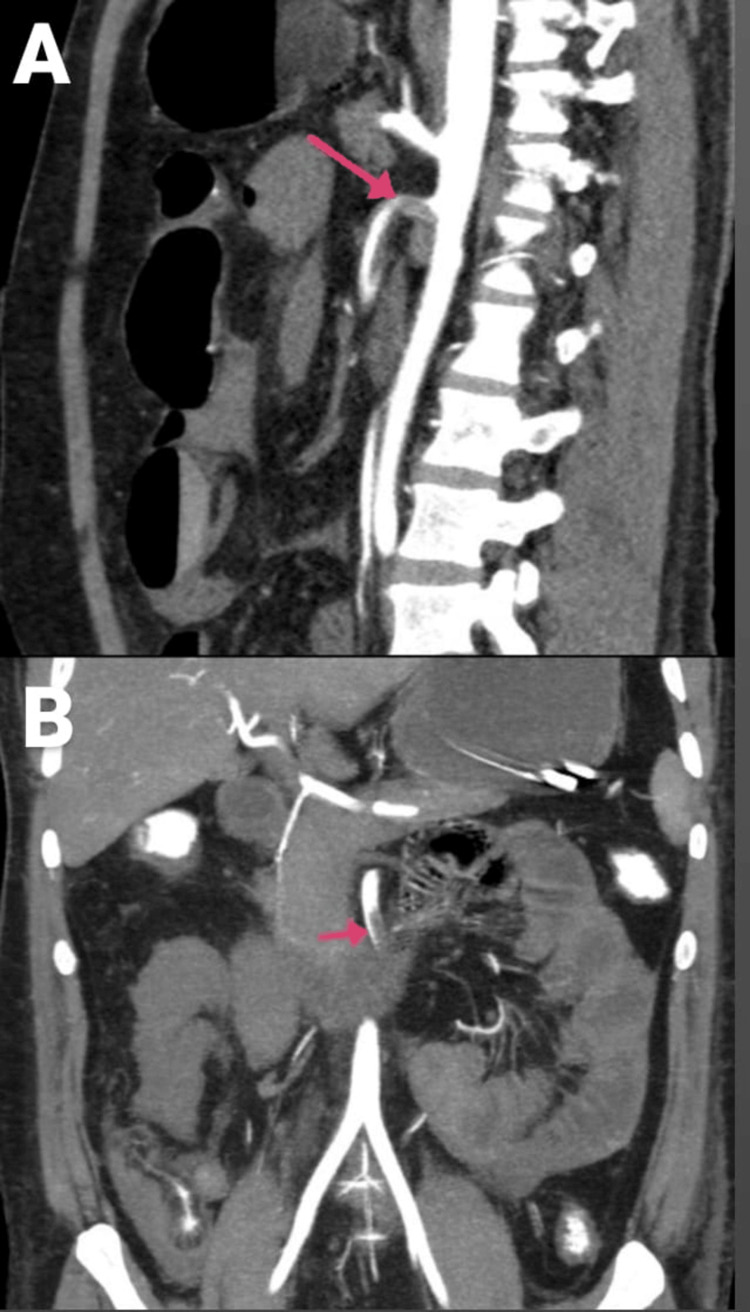
CECT of the abdomen and pelvis with angiography of the patient: (A) sagittal section and (B) coronal section (arrows showing the SMA thrombosis) CECT, contrast-enhanced computed tomography; SMA, superior mesenteric artery

A 34-year-old male presented with complaints of abdominal pain for four days. No complaints of nausea or vomiting were mentioned, and there were no comorbidities. The CECT of the abdomen and pelvis indicated partial filling defects in the SMA measuring 2 cm in length and positioned 1.5 cm from the aortic origin, causing a 50% narrowing (Figures 9A-9B, 10A-10B). No opacification of SMA was seen 5.6 cm from the origin, with no opacification of the jejuna and ileal branches on the right side, along with the right colic and ileocolic branches. Complete thrombosis of SMA was noted, accompanied by thrombosis in several small bowel branches, right colic, and ileocolic branches. Multiple dilated jejunal and ileal loops in the central and right sides of the abdomen showed poor mucosal enhancement and marked fat stranding, suggestive of bowel ischemia or infarction.

Intraoperative Findings

A gangrenous small bowel was seen from about 180 cm from the duodenojejunal flexure to about 100 cm distal to the ileocecal junction. Resection of the gangrenous bowel with SMA thrombectomy along with Ileostomy, as well as colostomy, was performed.

Postoperative Management

The patient suffered from septic shock with multiple febrile episodes postoperatively and was started on higher antibiotics and anticoagulation therapy; however, the patient succumbed three days postoperatively.

Case 5

**Figure 11:**
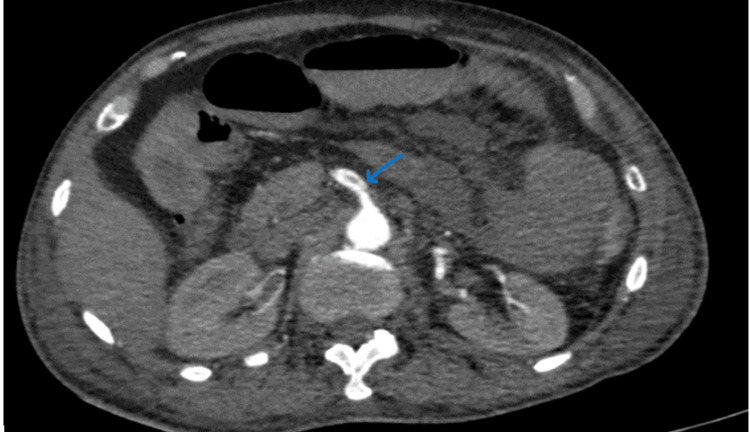
CECT of the abdomen and pelvis of the patient. CECT, contrast-enhanced computed tomography

A 44-year-old male had been complaining of pain in the abdomen for four days, bilious vomiting for four days, and Malena for four days. No comorbidities were noted. The patient tested positive for COVID-19. CECT of the abdomen and pelvis (Figure 11) showed a filling defect in the SMA at its origin, extending 2 cm in length with a thin rim of circumferential contrast enhancement, suggesting partial thrombosis. The distal segment was well opacified. Distal jejunal loops in the infraumbilical region extending to the hypogastrium showed decreased wall enhancement, suggesting early bowel ischemia.

Intraoperative Findings

**Figure 12:**
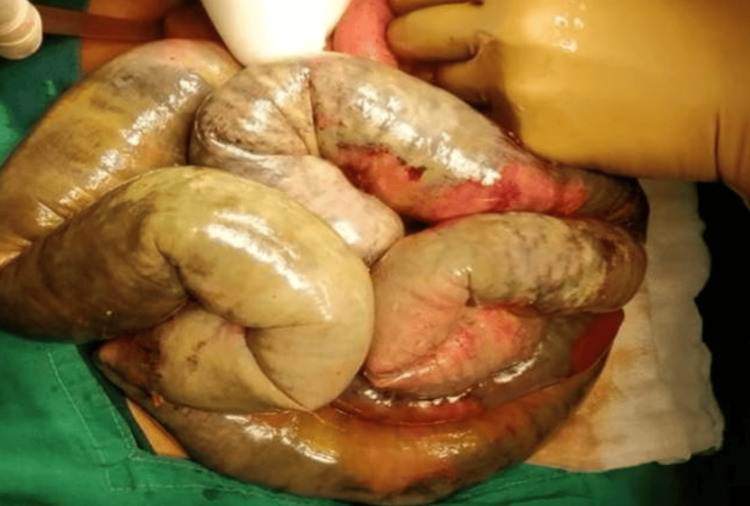
Intra-op photo of the gangrenous small bowel.

The gangrenous small bowel was seen from about 120 cm from the duodenojejunal flexure to about 50 cm proximal to the ileocecal junction (Figure 12). Resection of the gangrenous bowel, SMA thrombectomy, and ileoileal anastomosis were done.

Postoperative Management

The patient had no postoperative complications and was started on anticoagulation therapy and diet and then discharged. The patient had no complaints and was followed up for one year.

## Discussion

The jejunum, the ileum, and the colon are supplied mostly by the SMA [3]. SMA blockage from embolism causes 70% of instances of AMI, while thrombosis causes 30% of cases [1]. Abdominal emergencies like AMI are uncommon and typically necessitate broad intestine resection, have a high fatality rate, and, in individuals who survive, cause short-bowel syndrome [3].

AMI, particularly SMA occlusion, is a serious illness that necessitates immediate identification and treatment due to its high death rate of 60% to 80%. Knowing the harmful nature of both conditions separately, COVID-19 and acute SMA blockage appear to be an extremely fatal combo [5]. Less frequently, mesenteric arteries, the brain, and lower extremities have experienced thrombotic events. Because the S-type membrane protein of the virus ties up to ACE2, which is mostly seen in the endothelium, gut, lung, and liver, interleukin-6 (IL-6) production is stimulated by a reduction in angiotensin II breakdown, which has a pathogenic impact. Angiotensin II induces tissue factor and plasminogen activator type 1 inhibitor expression, which results in hypercoagulability. The lecithin route (C5b-9, C4d, and MASP2) is thought to be activated at the intestinal level, where the virus has been directly observed to be involved, leading to endothelial damage. The prevalence of abdominal symptoms is between 3% and 39%. According to reports from 2019, 1.9% to 4% of patients with COVID-19 have mesenteric ischemia. Mesenteric ischemia is a terrible condition; without early detection and treatment, the condition progresses to intestinal gangrene, which has significant rates of morbidity and fatality [6].

In addition to respiratory ailments, pneumonia is one of the organ damage conditions that COVID-19 is known to cause [7]. Most typical COVID-19 symptoms include fever, coughing, body aches, breathlessness, chest pain, headaches, diarrhea, and vomiting. However, thrombotic event-related secondary symptoms are also noted [8]. Symptoms and complications of COVID-19 include coagulopathy, which is frequently observed in acute infections like influenza. Due to this hypercoagulable state, conditions like pulmonary embolism and SMA thrombosis might develop [9].

COVID-19 can also cause cardiovascular, neurological, and ischemic problems. The underlying mechanism is currently not well known. Endotheliitis and hypercoagulability may be implicated. Even during the incubation period, the death rate for surgical patients is significant. To lessen the danger of transmission, comprehensive personal protection gear and customized solutions are essential [8].

The levels of D-dimer are the best test for detecting a thromboembolic consequence during COVID-19 sickness, which improves the prognosis. D-dimer is elevated in 36% of patients with COVID-19 (>0.9 mg/L). To prevent intestinal ischemia, portal hypertension, and exploratory surgery, which has significant postoperative comorbidity, risk factors must be identified, and anticoagulant medication should be started as soon as feasible [5,7].

Compared to patients with suspected embolism, those with suspected SMA thrombosis frequently require intestinal resection. At the origin of the SMA, where it is flush with the aorta, thrombotic occlusion develops and prevents collateral flow through the SMA's early branches [10].

It has been proposed that changes in the microcirculation are the cause of the coagulopathy brought on by SARS-CoV-2. One theory postulates that viral replication results in the infiltration of inflammatory cells into the endothelium, which leads to endothelial death and later microvascular prothrombotic events. The angiotensin-converting enzyme 2 (ACE2) receptors, present in the vascular endothelium along with the enterocytes of the small intestine, have also been shown to be activated by SARS-CoV-2, supporting the virus's microvascular thrombotic effects on the bowel. The majority of coagulopathies caused by COVID-19 present as pulmonary emboli; however, myocardial infarction (MI), stroke, arterial thrombosis, venous thromboembolism, and microvascular thrombosis have also been reported in some cases. AMI is rare, with a global incidence of less than 1%, but after COVID-19, it is of great concern to avoid unfavorable, maybe deadly, outcomes [4].

The interaction of hypercoagulability, endothelial dysfunction, hypoxia, and immobility may account for AMI in COVID-19, even though the precise pathophysiology is uncertain [1]. COVID-19 is connected to two diseases: (1) endotheliitis brought on by diffuse endothelial damage and infiltration of inflammatory cells and (2) a systemic hypercoagulable state brought on by hyperinflammation and hypercytokinemia. These features of patients with COVID-19 provide a plausible explanation for the production of an arterial occlusion [11].

According to some theories, thromboinflammation causes a cytokine storm, which boosts the coagulation processes that cause both minor and large vein thrombosis while also increasing IL-1, IL-6, IL-7, and tumor necrosis factor (TNF). SARS-CoV-2 has been seen to activate ACE2 receptors in several organs, including the intestine. This causes endothelial damage and thrombosis, which results in an ischemic process. VCAM-1, ICAM-1 activation, E-selectin, tissue factor, and plasminogen activator inhibitor 1 all prevent fibrinolysis. The coagulation parameters that are severity-related are activated partial thromboplastin time, prothrombin time, fibrin degradation products, D-dimer, and platelet count [7].

Both ischemic and non-ischemic factors contribute to the pathophysiology of the digestive manifestations observed in individuals with COVID-19. At the level of the gut wall, ACE2 receptors are present, and SARS-CoV-2 may infect enterocytes directly. Infected subjects had signs of the virus on their enteral walls and in their feces. The role of fecal viral ARN removal in the SARS-CoV-2 infection transmission chain is still not fully understood. On the other hand, bowel ischemia, leading to intestinal necrosis and perforation, was attributed to the disruption of the lung-gut axis and the procoagulation state induced by endothelial injury from SARS-CoV-2. It is crucial to identify and treat gastrointestinal ischemia as soon as possible [12].

Biomarkers such as serum lactate, which provide a general indication of tissue hypoperfusion, only show a significant increase in the presence of severe mesenteric damage. The two isomers are L-lactate, a general biomarker of anaerobic metabolism, and D-lactate, which is produced by the activities of gut flora. Increased D-lactate levels are more specific for mesenteric ischemia; however, findings from various studies are often contradictory. Interpreting the results in COVID-19 individuals may be challenging due to lactate and LDH being distinct risk factors for severe manifestations of the disease. Ischemic bowel disease should be one of the differential diagnoses in patients who suddenly develop abdominal pain and distension. Intestinal ischemia may be addressed with conservative methods if diagnosed in time [12].

When there is inconclusive clinical evidence, computed tomography angiography (CTA) and magnetic resonance angiography (MRA) are investigations that can help with the diagnosis process. These tests allow us to differentiate between non-thrombotic and thrombotic causes. Using an endovascular technique like percutaneous transluminal angioplasty and thrombolysis has a 90% success rate for lysis of the emboli in patients with SMA embolism (SMAE) [3]. Ottinger demonstrated an overall relationship between the prognosis, the location of the blockage, and the area of the infected regions. To make this theory clearer, SMA is divided into four components. The arterial origin represents the first tract, and the main trunk represents the second tract, which also includes the middle coronary artery (MCA). The third tract is the main trunk past the MCA's origin. The fourth tract reflects the SMA and its branches at their most remote points; a more significant infarction occurs when the SMA is blocked at its origin as opposed to when it is stopped further from the origin of some of its branches. The etiologic subgroups have an impact on the prognosis as well. In general, we can distinguish between thrombotic and non-thrombotic origins. Nonocclusive mesenteric ischemia is brought on by low-flow conditions. The three signs of thrombotic illness are arterial embolism, arterial thrombosis, and mesenteric venous thrombosis. Poor symptoms, signs, and vague laboratory testing are a few of the things that contribute to the diagnosis being delayed [12].

Low-molecular-weight heparin is advised as the first-line drug in patients with COVID-19 who require anticoagulation because it has greater stability than heparin during cytokine storms and a lower chance of interaction with antiviral medication than anticoagulants. Heparin, in addition to serving as an anticoagulant, has been noted by certain researchers to have anti-inflammatory properties in cases of severe COVID-19 infection. Heparin lessens inflammation by decreasing neutrophil activity, inflammatory mediator expression, and vascular smooth muscle cell proliferation. For patients with mild-to-moderate COVID-19, thromboprophylaxis with enoxaparin may also be recommended. Drugs like anti-complement and IL-1 receptor antagonists can also be considered. IL-1 significantly contributes to endothelial dysfunction, inflammation, and thrombi development by encouraging the production of thromboxane A2 and thromboxane B2 [13].

In the majority of SMA blockage cases, necrotic bowel resection is required to preserve life [11]. The histology of patients with COVID-19 revealed microthrombi and fibrinogen deposits, which suggested a direct effect on the coagulation cascade [14].

The second-look laparotomy is still the gold standard for determining whether or not the gut will continue to function because it is the only procedure for removing infarcted intestinal tracts. After acute mesenteric ischemia, the survival rates for the three etiological groups varied. The mortality rate after surgery for arterial embolism and venous thrombosis has decreased, although it is still high for thrombosis in the arterial blood vessels and nonocclusive ischemia [15].

## Conclusions

Doctors have significant clinical challenges as a result of the thromboembolic manifestations of the virus's unexpected and deadly nature, such as AMI. Early detection of AMI and the identification of individuals at highest risk are essential. The high morbidity and mortality associated with AMI calls for further study on prophylactic anticoagulation therapy in COVID-19-positive individuals. Physicians should be cautious about SMA occlusion when patients with COVID-19 complain of severe stomach pain since the condition could be caused by the virus's secondary microangiopathy, which directly impacts the coagulation cascade. Because the illness can be fatal, early detection and treatment are essential.

## References

[1] Maniar Y, Hashmi H, Silverstein J, Chung C, Kella V, Goparaju A (2022). Superior mesenteric artery thrombosis in a patient with COVID-19 pneumonia and Clostridium difficle. J Surg Case Rep.

[2] Kozuch PL, Brandt LJ (2005). Review article: diagnosis and management of mesenteric ischemia with an emphasis on pharmacotherapy. Aliment Pharmacol Ther.

[3] Romano N, Prosperi V, Basili G (2011). Acute thrombosis of the superior mesenteric artery in a 39-year-old woman with protein-S deficiency: a case report. J Med Case Rep.

[4] Segovia FD, Ream S, Dang T, Chaganti BT, Ortega AJ, Rhee S, Borges JC (2022). COVID-19-associated superior mesenteric artery thrombosis and acute intestinal ischemia. Cureus.

[5] Fad S., Hicham K., Nizar E. (2022). Acute mesenteric thrombosis in COVID-19: a case report. Open Access Library J.

[6] Kurland B, Brandt LJ, Delany HM (1992). Diagnostic test for intestinal ischemia. Surg Clin North Am.

[7] Romero MD, Cárdenas AM, Fuentes AB, Barragán AA, Gómez DB, Jiménez MT (2021). Acute mesenteric arterial thrombosis in severe SARS-Co-2 patient: a case report and literature review. Int J Surg Case Rep.

[8] Bianco F, Ranieri AJ, Paterniti G, Pata F, Gallo G (2020). Acute intestinal ischemia in a patient with COVID-19. Tech Coloproctol.

[9] de Barry O, Mekki A, Diffre C, Seror M, El Hajjam M, Carlier RY (2020). Arterial and venous abdominal thrombosis in a 79-year-old woman with COVID-19 pneumonia. Radiol Case Rep.

[10] Björck M, Acosta S, Lindberg F, Troëng T, Bergqvist D (2002). Revascularization of the superior mesenteric artery after acute thromboembolic occlusion. Br J Surg.

[11] Sukegawa M, Nishiwada S, Terai T (2022). Acute superior mesenteric artery occlusion associated with COVID-19 pneumonia: a case report. Surg Case Rep.

[12] Ottinger LW (1978). The surgical management of acute occlusion of the superior mesenteric artery. Ann Surg.

[13] Serban D, Tribus LC, Vancea G (2021). Acute mesenteric ischemia in COVID-19 patients. J Clin Med.

[14] Aryal S, Bhattarai V, Sharma S (2023). SARS-COV-2-related superior mesenteric artery thrombosis resulting in pneumatosis intestinalis complicated by pneumatosis portalis in a young male: a case report. Ann Med Surg (Lond).

[15] Schoots IG, Koffeman GI, Legemate DA, Levi M, van Gulik TM (2004). Systematic review of survival after acute mesenteric ischaemia according to disease aetiology. Br J Surg.

